# *Lactobacillus reuteri* SBC5-3 suppresses TNF-α-induced inflammatory responses via NF-κB pathway inhibition in intestinal epithelial cells

**DOI:** 10.3389/fmicb.2025.1573479

**Published:** 2025-07-08

**Authors:** Shiyu Chen, Tiannian Hu, Le Xu, Jiqin Li, Chen Liu, Bangquan Zeng, Qiuye Lin, Zhenhui Cao

**Affiliations:** ^1^Faculty of Animal Science and Technology, Yunnan Agricultural University, Kunming, China; ^2^Yunnan Provincial Key Laboratory of Animal Nutrition and Feed Science, Kunming, China; ^3^Yunnan Center for Animal Disease Control and Prevention, Kunming, China; ^4^School of Ethnic Medicine, Yunnan Minzu University, Kunming, China

**Keywords:** *Lactobacillus reuteri*, nuclear factor-κB signaling, tumor necrosis factor-alpha, anti-inflammatory, intestinal epithelial cells

## Abstract

The widespread misuse of antibiotics in livestock production has raised growing concerns about antimicrobial resistance and residue contamination. These challenges have led to global restrictions on the use of antibiotic growth promoters (AGPs). However, the ban on AGPs has made the management of intestinal inflammation significantly more difficult, highlighting the urgent need for safe and effective alternatives. Lactic acid bacteria (LAB), known for their immunomodulatory properties, have emerged as promising candidates, though their anti-inflammatory mechanisms remain poorly understood. This study investigated the anti-inflammatory effects and underlying molecular mechanisms of a porcine-derived *Lactobacillus reuteri* (*L. reuteri*) strain, SBC5-3. Using tumor necrosis factor-alpha (TNF-α)-induced inflammatory models in the HT-29 cells, we employed RNA sequencing (RNA-seq) combined with Western blot analyses to systematically explore the modulation of inflammatory signaling pathways. RNA-seq demonstrated that *L. reuteri* SBC5-3 downregulated 9 out of 14 TNF-α-induced genes associated with the TNF and/or nuclear factor kappa B (NF-κB) signaling pathways, including *BIRC3*, *PTGS2*, *CCL20*, *TNFAIP3*, *LTB*, *CXCL1*, *CXCL10*, *IL-8*, and *CSF1*. Specifically, *L. reuteri* SBC5-3 downregulated key genes in the NF-κB pathway, such as *TAK1*, *IKK*α, and *NFKB1*, while upregulating *NFKBIA*. Concurrent transcriptional suppression occurred in mitogen-activated protein kinase (MAPK) signaling pathway components *ERK* and *JNK*. Western blot analysis further confirmed attenuation of phosphorylation involving TAK1, IKKα/β, and IκBα following SBC5-3 treatment. The collective data demonstrate that immunomodulation mediated by *L. reuteri* SBC5-3 involves inhibiting IκBα degradation, which is known to be essential for the nuclear translocation of the p50/p65 heterodimer, thereby suggesting inhibition of TNF-α-induced NF-κB nuclear translocation. These results position *L. reuteri* SBC5-3 as a viable therapeutic agent for inflammation modulation through targeted intervention in NF-κB and MAPK signaling pathways.

## 1 Introduction

For decades, antibiotics have been widely incorporated into livestock feed as AGPs to improve production efficiency, suppress enteric pathogens, and reduce production costs (Liu et al., 2005; Brown et al., 2017). The growth-enhancing mechanism of AGPs primarily involve modulation of intestinal inflammatory responses and reduction of immunological burden (Niewold, 2007). However, with increasing concerns regarding antibiotic residues and resistance stemming from overuse, there has been a growing trend to prohibit AGPs in animal feed in many countries and regions (Miyakawa et al., 2024). While crucial for addressing antibiotic misuse and related complications, it has introduced novel challenges. Restricting AGPs in livestock nutrition exacerbates management of intestinal inflammation induced by pathogens, antinutritional components in feed, and environmental factors, ultimately compromising both animal welfare and farming efficacy (Adedokun and Olojede, 2019). Development of viable antibiotic alternatives now constitutes a priority challenge for sustainable agriculture systems.

LAB, indigenous gut probiotics with multifunctional benefits, represent viable antibiotic alternatives in animal husbandry (Vieco-Saiz et al., 2019). Previous studies has demonstrated LAB-mediated improvements in livestock feed utilization efficiency, suppression of enteropathogen colonization, and maintenance of intestinal redox homeostasis (Wang et al., 2016; Bell et al., 2022; Li et al., 2024). Emerging evidence highlights immunoregulatory properties across numerous *Lactobacilli* strains. For instance, *L. plantarum* MTCC 9483 was shown to differentially regulate inflammatory mediators in Caco-2 cells under oxidative stress, lipopolysaccharides (LPS) induction, and pathogen invasion, suppressing pro-inflammatory cytokines (*TNF-*α, interleukin *[IL]-1*α, *IL-1*β, *IL-6*, and *IL-8*) while simultaneously upregulating anti-inflammatory genes (*TNF-*β*1*, *IL-*4, and *IL-10*) (Devi et al., 2018). Experimental evidence (Devi et al., 2018) established that dietary supplementation with *L. johnsonii* Jlus66 diminished pro-inflammatory cytokines (TNF-α, *IL-1*β, and *IL-6*) levels in dextran sulfate sodium-induced colitis mice and inhibited the MAPK and NF-κB signaling pathways. Although LAB-mediated anti-inflammatory effects are extensively documented, mechanistic elucidation of these immunomodulatory interactions remains incomplete.

The inflammatory process involves intricate cross-talk among multiple signaling pathways, requiring systematic analysis through modern technological approaches. RNA sequencing (RNA-seq) technology, distinguished by high-throughput capacity, analytical sensitivity, and cost-efficiency, has emerged as an effective methodology for elucidating complex regulatory networks in biological systems (Jackson et al., 2024). Hou et al. (2019) employed RNA-seq to delineate antioxidant pathway activation in *L. rhamnosus* GG and *L. plantarum* J26 following hydrogen peroxide (H_2_O_2_)-induced conditions in Caco-2 intestinal models. Similarly, Cheng et al. (2024) applied RNA-seq profiling to reveal *L. reuteri* IMAUJBC1-mediated attenuation of diet-induced hyperlipidemia in murine models through peroxisome proliferator-activated receptor (PPAR) signaling pathway. These investigations collectively validate RNA-seq as an indispensable tool for mechanistic exploration of probiotic mechanisms and host-probiotic interactions.

Our previous investigations identified *L. reuteri* SBC5-3, a strain isolated from porcine intestinal specimens in Yunnan Province, China, demonstrating significant probiotic characteristics. This strain exhibits key probiotic traits such as gastrointestinal tract tolerance, strong adhesion, and antimicrobial efficacy. Notably, preliminary *in vitro* experiments indicatedthat co-culture with SBC5-3 substantially reduced TNF-α-stimulated IL-8 secretion in HT-29 cells, implicating its anti-inflammatory capacity (Bao et al., 2023). RNA-seq was employed to profile transcriptional alterations in TNF-α-induced HT-29 cells following co-cultured with *L. reuter*i SBC5-3, complemented by Western blot analysis to quantify phosphorylation status of critical NF-κB pathway mediators: transforming growth factor-β-activated kinase 1 (TAK1), inhibitor kappa B kinase (IKK) α/β, and inhibitor of kappa B alpha (IκBα). This investigation seeks to elucidate the molecular basis underlying *L. reuteri* SBC5-3-mediated suppression of TNF-α-induced inflammation in HT-29 cells, thereby providing foundational insights essential for advancing novel anti-inflammatory formulations in animal nutrition.

## 2 Materials and methods

### 2.1 Culture and preparation of *L. reuteri* SBC5-3 suspension

SBC5-3 was originally isolated by our research team from fecal samples of free-range Saba pigs in Chuxiong, Yunnan, China, and cryopreserved at –80°C. Preliminary whole-genome sequencing of strain SBC5-3 revealed that its average nucleotide identity (ANI) values exceeded the 95% species threshold when compared with five representative *L. reuteri* strains (Supplementary Figure S1), thereby confirming its taxonomic classification as *L. reuteri*. A sterile inoculation loop was used to transfer a minimal aliquot of the cryostock onto de Man Rogosa Sharpe agar (MRS; Merck, Darmstadt, Germany), followed by aerobic incubated at 37°C for 48 h. Distinct colonies were subcultured into liquid MRS medium, homogenized, and cultured under standard conditions (37°C, 16 h). Subsequently, the culture was augmented by inoculating 1% (v/v) of the previous culture into fresh medium, with subsequent incubation for 8 h under identical parameters. The bacterial cells were harvested by centrifugation at 4,500 rpm for 10 min at 4°C and washed three times with phosphate-buffered saline. The pellet was subsequently resuspended in Roswell Park Memorial Institute-1640 (RPMI-1640) medium (VivaCell, Shanghai, China), and the concentration was adjusted to 1 × 10^8^ colony-forming units (CFU)/mL, as determined by *OD*_600_ measurements and a CFU calibration curve.

### 2.2 Culture of HT-29 cells

The human colon adenocarcinoma HT-29 cell line (Catalog number: KCB200508YJ) was kindly provided by the Kunming Institute of Zoology, Chinese Academy of Sciences. Cells were routinely cultured in RPMI-1640 medium supplemented with 10% fetal bovine serum (VivaCell, Shanghai, China), and 1% penicillin-streptomycin (Biological Industries, Kibbutz Beit Haemek, Israel). The cells were maintained in 25-cm^2^ cell culture flasks. Incubation was performed at 37°C in a humidified 5% CO_2_ atmosphere, with medium replacement every 48-h and weekly subculturing at 1:3 ratio. Cells from passages 3–5 were utilized for the subsequent experiments.

### 2.3 Effect of *L. reuteri* SBC5-3 on TNF-α-induced transcriptomic changes in cells

#### 2.3.1 Experimental groups and treatment

TNF-α (PeproTech, NJ, United States) stock solution (10 μg/mL) was prepared by reconstituting 50 μg of lyophilized powder in 0.5 mL sterile, nuclease-free water with repeated pipetting, followed by PBS dilution to a final volume of 5 mL. The solution was aliquoted into sterile, nuclease-free 1.5mL microcentrifuge tubes and stored at −20°C until use.

HT-29 cells were seeded at 1.5 × 10^6^ cells/well in a six-well plate. After reaching 80% confluence, the medium was discarded, and the cells were washed thrice with pre-warmed RPMI-1640 medium. The cells were subsequently prepared for treatment. The experiment comprised four groups, each containing four biological replicates, as follows:

Control group (CON): Cells were cultured in 2 mL RPMI-1640 medium for 19 h.

TNF-α group (TNF): Cells were cultured in 2 mL of RPMI-1640 medium for 16 h, then treated with 10 μL of 10 μg/mL TNF-α stock solution (final concentration: 50 ng/mL) for 3 h.

SBC5-3 + TNF-α group (SBC + TNF): Cells were pre-incubated for 16 h in 2 mL of RPMI-1640 medium containing *L. reuteri* SBC5-3 (1 × 10^8^ CFU/mL); followed by treatment with 10 μL of 10 μg/mL TNF-α stock solution (final concentration: 50 ng/mL) for 3 h.

SBC5-3 group (SBC): Cells were cultured in 2 mL of RPMI-1640 medium containing *L. reuteri* SBC5-3 (1 × 10^8^ CFU/mL) for 19 h.

All incubations were conducted at 37°C in a humidified 5% CO_2_ incubator. Post-treatments, total RNA was extracted using the RNA Simple Total RNA Extraction Kit (DP419, Tiangen Biotech, Beijing, China) following manufacturer’s instructions. RNA purity was assessed with a Microvolume Spectrophotometer (IMPLEN, Munich, Germany), while agarose gel electrophoresis was used to verify RNA quality. The extracted RNA was subsequently stored at –80°C for further analysis.

#### 2.3.2 RNA Sequencing and differential expression analysis

Total RNA samples stored at –80°C were processed for ribosomal RNA (rRNA) depletion and strand-specific library construction. After the quality control of the libraries, transcriptomic sequencing was performed utilizing the Illumina NovaSeq™ 6000 platform (Illumina, San Diego, CA, United States). Raw sequences were processed using Cutadapt to eliminate low-quality reads, resulting in high-quality data. The resultant clean data were aligned to the human reference genome utilizing HISAT2, and location-specific information was annotated for genes or genomic regions, along with sequence characteristics unique to the sequencing samples.

Gene expression was quantified by calculating the fragments per kilobase of transcript per million mapped reads (FPKM) for each gene. The number of reads mapped to each gene was counted. Differential expression analysis was conducted to calculate the fold changes and assess statistical significance. Differentially expressed genes (DEGs) were identified using the criteria of false discovery rate (FDR)-adjusted *p*-value (*q*-value) < 0.05 and Log_2_ (fold change) > 1. These DEGs underwent subsequent Kyoto encyclopedia of genes and genomes (KEGG) pathway enrichment analysis using the same *q*-value < 0.05 for statistical significance.

### 2.4 Real-time quantitative polymerase chain reaction (RT-qPCR) validation of DEGs

A subset of significant DEGs identified in the RNA-seq were selected for RT-qPCR validation to validate the reliability of the RNA-seq data. RT-qPCR was used to measure mRNA abundance of the following genes: *ACTB*, *IL-8*, *IL-1*β, *CCL20*, *CXCL10*, *NFKB1*, *NFKBIA*, *PTGS2*, and *TNFAIP3*. Shanghai Generay Biotechnology Co., Ltd. synthesized primers for RT-qPCR, as detailed in Supplementary Table S1.

PCR amplification was performed under the following thermal cycling conditions: initial denaturation at 94°C for 5 min, followed by 40 cycles of 94°C for 15 s, 60°C for 45 s, and a final elongation step at 54°C for 10 min. A negative control without the target gene was included in the PCR reactions. Fluorescence was measured at 60°C during extension phases, followed by melting curve analysis to confirm the specificity of amplification. The 2^−ΔΔCt^ method (Livak and Schmittgen, 2001) was used for data analysis.

### 2.5 Effect of *L. reuteri* SBC5-3 on TNF-α-induced activation of the NF-κB inflammatory signaling pathway in cells

According to the grouping method outlined in section 2.3.1, four groups (CON, TNF-α, SBC5-3, and SBC5-3 + TNF-α) were cultured for 16 h. Following the initial culture period, the cells were subjected to specific treatments based on their group assignments.

In the CON and SBC5-3 groups, cells were further cultured without any additional treatment for 15, 30, 45, and 60 min.

In the TNF-α and SBC5-3 + TNF-α groups, 10 μL of 10 μg/mL TNF-α was added to achieve a final concentration of 50 ng/mL. The cultures were then maintained for 15, 30, 45, and 60 min.

After the designated treatment times, cells were harvested for protein extraction. The cells were lysed in a buffer containing RIPA lysis buffer (Solarbio, Beijing, China) and protease inhibitors, homogenized through pipetting, supplemented with 10% sodium dodecyl sulfate, and incubated at 4°C for 20 min. The lysates were heated in boiling water for 15 min, followed by centrifugation at 12,000 rpm for 5 min at 4°C, after which supernatants were collected. Protein concentration was measured with a bicinchoninic acid assay kit (Thermo Fisher Scientific, MA, United States). Equal protein quantities (10 μg) underwent Sodium Dodecyl Sulfate-Polyacrylamide Gel Electrophoresis separation before transfer onto nitrocellulose membranes (GE Healthcare, IL, United States). Membrane blocking was performed for 1 h at room temperature using 5% non-fat dry milk (BD Difco) in Tris-buffered saline with Tween 20. Subsequent to blocking, membranes were subjected to overnight incubation at 4°C with primary antibodies targeting TAK1 and p-TAK1 (Biorbyt, Cambridge, United Kingdom); IKKα/β (Abcam, Cambridge, United Kingdom); p-IKKα/β and p-IκBα (Cell Signaling Technology, MA, United States); IκBα and β-actin (PeproTech, NJ, United States). Following washing procedures, membranes were treated with horseradish peroxidase (HRP)-conjugated goat anti-rabbit IgG (PeproTech, NJ, United States). Visualization of immunoreactive bands was achieved using a gel imaging system (Bio-Rad Laboratories, CA, United States) and quantified with ImageJ software (NIH, Bethesda, MD, United States).

### 2.6 Data analysis

Protein expression data were calculated as the ratio of the intensity of the target protein band to that of the housekeeping protein band. Initial data organization was performed using Excel 2019 (Microsoft Corporation, Redmond, WA, United States), followed by statistical analysis using SPSS 25.0 software (IBM, Armonk, NY, United States). Statistical comparisons were made via Student’s *t*-test, with results reported as the mean ± standard deviation. Statistical significance was set at *P* < 0.05.

## 3 Results

### 3.1 Effect of *L. reuteri* SBC5-3 on the transcriptome of TNF-α-induced cells

#### 3.1.1 Statistical analysis of transcriptome sequencing data

**FIGURE 1:**
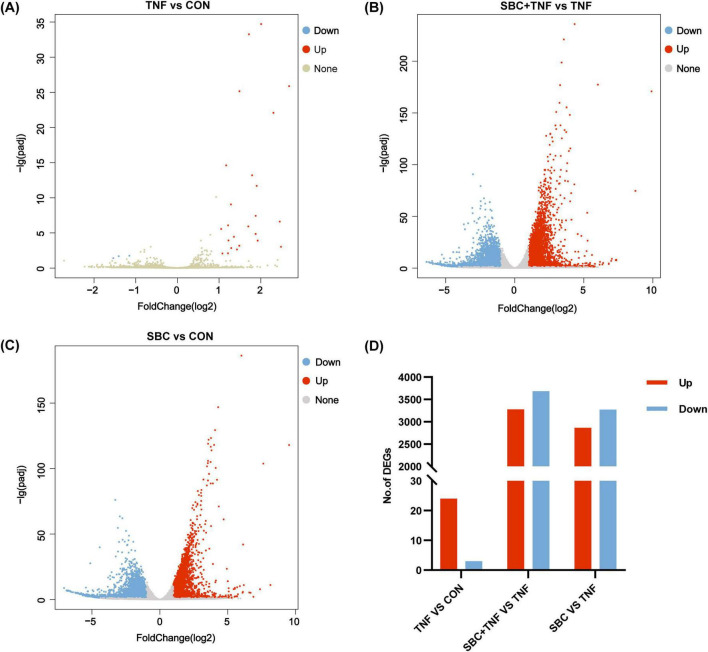
Volcano plots of DEGs between groups: **(A)** TNF versus CON; **(B)** SBC + TNF versus TNF. **(C)** SBC versus CON. **(D)** Summary of DEGs. Red represents upregulated DEGs, blue represents downregulated DEGs, and gray represents DEGs without significant changes. *n* = 4 replicates/group.

Figures 1A–D illustrate the expression of significant DEGs between the comparison groups. In the TNF-α versus CON group, 27 DEGs were detected, comprising 24 upregulated and 3 downregulated genes. The SBC5-3 versus CON comparison revealed 6,132 DEGs. with 2,863 upregulated and 3,269 downregulated transcripts. For the SBC5-3 + TNF-α versus TNF-α comparison, 6,961 DEGs were identified, including 3,276 upregulated and 3,685 downregulated genes.

#### 3.1.2 KEGG pathway enrichment analysis of DEGs

**FIGURE 2:**
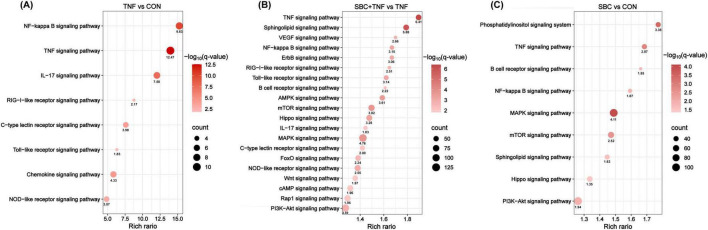
KEGG pathway enrichment analysis of signaling and immune-related pathways between groups: **(A)** TNF versus CON. **(B)** SBC + TNF versus TNF. **(C)** SBC versus CON. *n* = 4 replicates/group.

Functional annotation of relevant sequences was performed utilizing KEGG analysis. As shown in Figure 2A, eight signaling and immune-related pathways demonstrated significant enrichment in the TNF versus CON comparison, particularly NF-κB, TNF, and IL-17 signaling pathways. Notably, the NF-κB signaling pathway displayed the highest enrichment rate, indicating that TNF-α may induce the inflammatory response in HT-29 cells through NF-κB signaling. In contrast, the SBC5-3 + TNF versus CON comparison revealed enrichment of 20 signaling and immune-related pathways, including TNF, sphingolipid, VEGF, and NF-κB signaling pathways (Figure 2B). Similarly, nine pathways were enriched in the SBC versus CON comparison (Figure 2C), including phosphatidylinositol signaling, TNF, B cell receptor, and NF-κB signaling.

**FIGURE 3:**
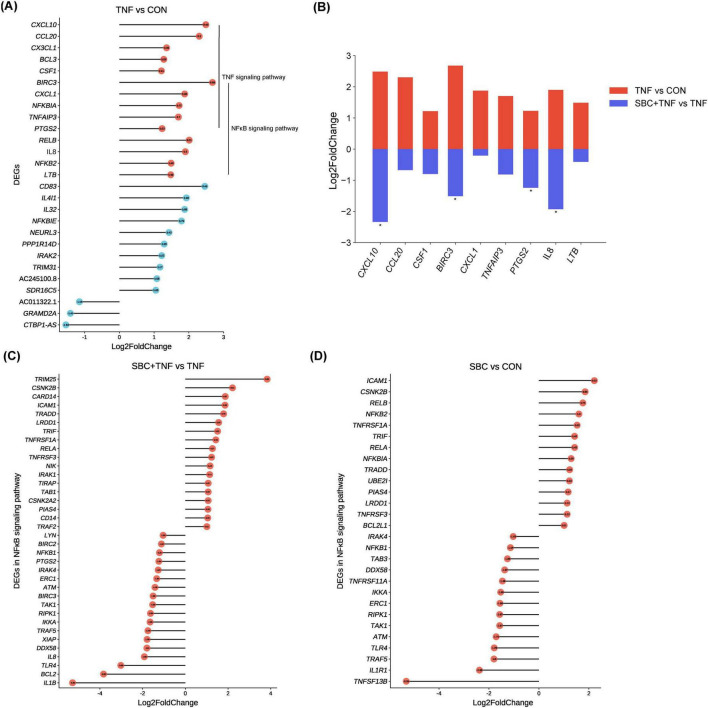
Changes in DEGs between groups: **(A)** Changes in all DEGs in the TNF versus CON group. **(B)** Changes in gene expression induced by TNF-α in HT-29 cells co-cultured with *L. reuteri* SBC5-3. **(C)** Changes in DEGs in the NF-κB signaling pathway in the SBC + TNF versus TNF group. **(D)** Changes in DEGs in the NF-κB signaling pathway in the SBC versus CON group. *n* = 4 replicates/group; In panel **(B)**, all genes shown in red for the TNF vs. CON comparison are DEGs, while for the SBC + TNF vs. TNF comparison, the genes marked with an asterisk “*” are DEGs.

Analysis of pathway enrichment revealed significant involvement of both NF-κB and TNF signaling pathways across all three experimental comparisons, suggesting potential modulation of TNF-α-induced inflammatory responses by *L. reuteri* SBC5-3 through these molecular pathways. In the TNF versus CON group, DEGs analysis identified 27 significantly altered genes in HT-29 cells following TNF-α treatment, comprising 24 upregulated and 3 downregulated transcripts (Figure 3A). Among these 27 DEGs, 14 were functionally associated with TNF and/or NF-κB signaling pathways, all exhibiting upregulated expression, which confirms successful induction of inflammatory response by TNF-α in HT-29 cells. Importantly, administration of *L. reuteri* SBC5-3 resulted in downregulation of 9 out of these 14 inflammation-related genes, namely C-X-C motif chemokine ligand (*CXCL*) 1, *CXCL10*, *IL-8*, C-C motif chemokine ligand 20 (*CCL20*), baculoviral IAP repeat containing 3 (*BIRC3*), prostaglandin-endoperoxide synthase 2 (*PTGS2*), tumor necrosis factor alpha induced protein 3 (*TNFAIP3*), lymphotoxin beta (*LTB*), and colony stimulating factor 1 (*CSF1*), with *IL-8*, *CXCL10*, *BIRC3*, and *PTGS2* exhibited significant downregulation (*P* < 0.05) (Figure 3B). Functional analysis identified predominant involvement of these genes in the NF-κB signaling pathway, where multiple components function as downstream effectors. This observation implies that *L. reuteri* SBC5-3 potentially regulates inflammation responses through modulation of NF-κB signaling cascade. This finding justify additional research to elucidate the precise mechanisms by which SBC5-3 influences gene expression within the NF-κB signaling pathway.

Analysis revealed 36 DEGs (18 upregulated and 18 downregulated) in the NF-κB signaling pathway when comparing SBC + TNF versus TNF conditions (Figure 3C). Similarly, the SBC versus CON comparison showed 29 DEGs (14 upregulated and 15 downregulated) within the same pathway (Figure 3D). Notably, *TAK1*, *IKKa*, and *NFKB1* expression exhibited significant downregulation in both experimental comparisons, representing key components of the NF-κB signaling cascade. Additionally, the SBC + TNF versus TNF comparison demonstrated reduced expression of toll-like receptor 4 (*TLR4*) along with critical MAPK signaling pathway components, specifically extracellular regulated protein kinases (*ERK*) and c-Jun N-terminal kinase (*JNK*) (Supplementary Table S2). Furthermore, the SBC versus CON comparison revealed upregulated expression of *NFKBIA*, a negative regulator of NF-κB signaling. These observations indicate that *L. reuteri* SBC5-3 may differentially modulate TNF-α-induced inflammatory response in HT-29 cells through distinct molecular mechanisms.

#### 3.1.3 Validation of transcriptome results by RT-qPCR

**FIGURE 4:**
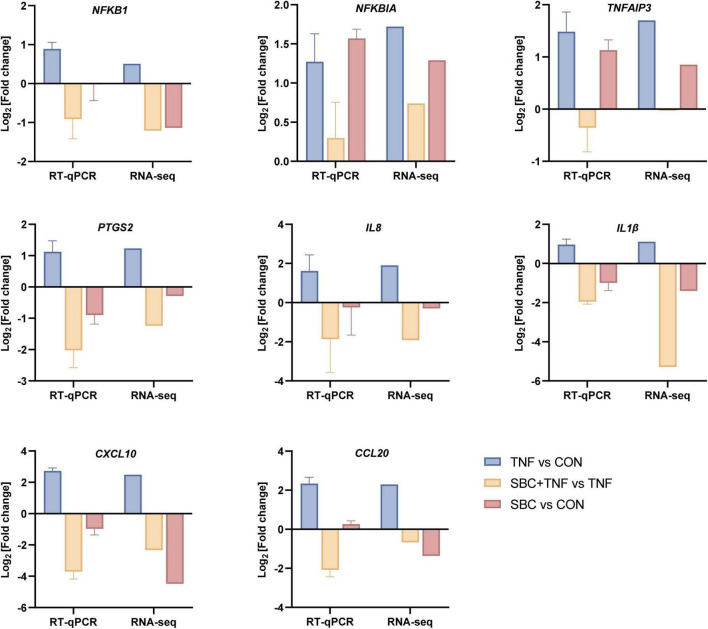
RT-qPCR validation of transcriptome DEG results. RT-qPCR data are presented as the mean ± SD. *n* = 4 replicates/group.

For validation of transcriptomic findings, eight genes from the NF-κB signaling pathway (*IL-8*, *IL-1*β, *CXCL10*, *CCL20*, *NFKB1*, *NFKBIA*, *TNFAIP3*, and *PTGS2*) were selected for RT-qPCR analysis. Figure 4 illustrate that the expression patterns of these genes observed in the DEG analysis were consistent with the trends detected by RT-qPCR. This correlation confirms the reliability of the transcriptome data.

### 3.2 *L. reuteri* SBC5-3 modulates NF-κB signaling pathway activation in TNF-α-induced cells

#### 3.2.1 Effect of *L. reuteri* SBC5-3 on phosphorylation of upstream kinases in the NF-κB Pathway in TNF-α-induced cells

**FIGURE 5:**
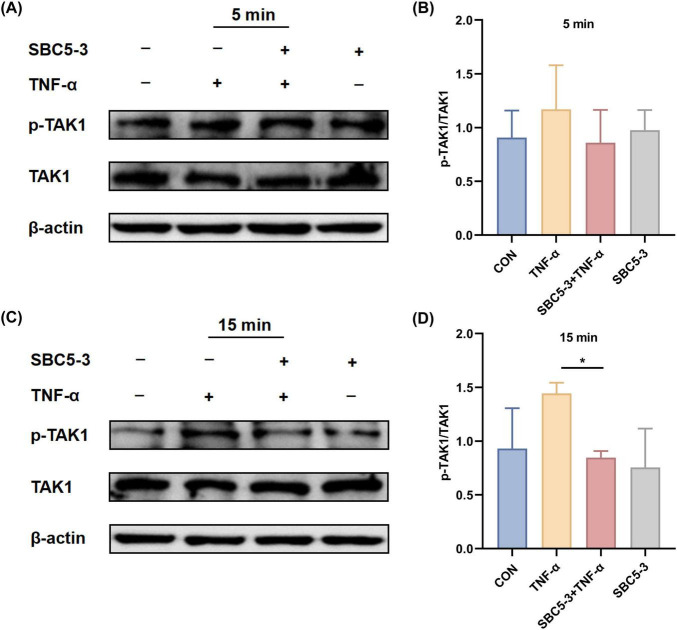
Effect of *L. reuteri* SBC5-3 on TNF-α-induced TAK1 phosphorylation in HT-29 cells. **(A,B)** TNF-α treatment for 5 min. **(C,D)** TNF-α treatment for 15 min. The “*” indicates a significant difference (*P* < 0.05); *n* = 4 replicates/group.

Quantitative analysis revealed an increased p-TAK1/TAK1 ratio in HT-29 cells at 5 and 15 min after TNF-α treatment compared to CON group (*P* > 0.05; Figure 5). In contrast, the SBC5-3 group showed no significant difference in the p-TAK1/TAK1 ratio at these time points (*P* > 0.05). When compared to the TNF-α group, the p-TAK1/TAK1 ratio in the SBC5-3 + TNF-α group decreased at 5 min (*P* > 0.05) and was significantly reduced at 15 min (*P* < 0.05). These data indicate that 15-min TNF-α exposure enhances TAK1 phosphorylation in HT-29 cells, while 16-h pre-incubation with *L. reuteri* SBC5-3 effectively mitigates this TNF-α-induced TAK1 phosphorylation response.

#### 3.2.2 Effect of *L. reuteri* SBC5-3 on TNF-α-induced NF-κB pathway IκB kinase activation in cells

**FIGURE 6:**
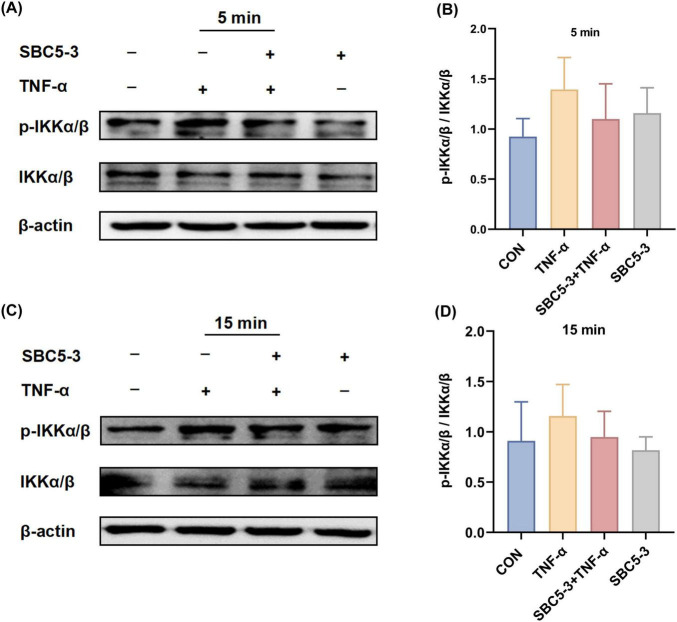
Effect of *L. reuteri* SBC5-3 on TNF-α-induced phosphorylation of IKKα/β in HT-29 cells. **(A,B)** TNF-α treatment for 5 min. **(C,D)** TNF-α treatment for 15 min. *n* = 4 replicates/group.

The effect of *L. reuteri* SBC5-3 on TNF-α-induced phosphorylation of IκB kinase IKKα/β in HT-29 cells is as follows. Compared to the CON group, TNF-α stimulation induced an increasing trend in the p-IKKα/β to IKKα/β ratio at 5 and 15 min (*P* > 0.05; Figure 6). In the SBC5-3 group, no significant changes were observed in the p-IKKα/β to IKKα/β ratio at these time points (*P* > 0.05). Notably, co-treatment with SBC5-3 + TNF-α demonstrated a decreasing trend in the p-IKKα/β to IKKα/β ratio at 5 and 15 min (*P* > 0.05). These results indicate that TNF-α promotes IκB kinase activation through enhanced IKKα/β phosphorylation, while 16-h pre-incubation with *L. reuteri* SBC5-3 suppresses this TNF-α-induced IκB kinase activation and reduces IKKα/β phosphorylation.

#### 3.2.3 Effect of *L. reuteri* SBC5-3 on phosphorylation and degradation of NF-κB inhibitor IκBα in TNF-α-induced cells

**FIGURE 7:**
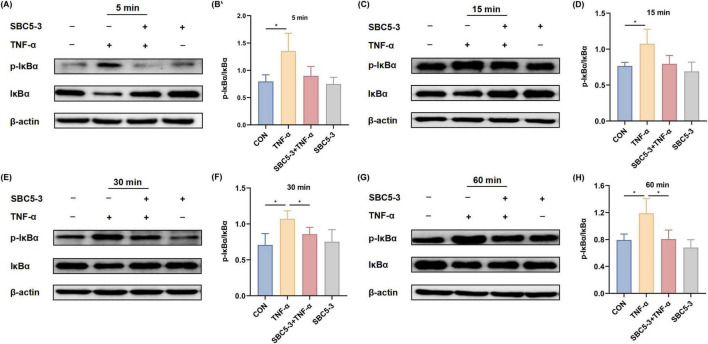
Effect of *L. reuteri* SBC5-3 on TNF-α-induced phosphorylation of IκBα in HT-29 cells. **(A,B)** TNF-α treatment for 5 min. **(C,D)** TNF-α treatment for 15 min. **(E,F)** TNF-α treatment for 30 min. **(G,H)** TNF-α treatment for 60 min. The “*” indicates a significant difference (*P* < 0.05); *n* = 4 replicates/group.

The effect of *L. reuteri* SBC5-3 on TNF-α-induced degradation and phosphorylation of the NF-κB inhibitor IκBα in HT-29 cells is as follows. TNF-α stimulation significantly elevated the p-IκBα/IκBα ratio relative to CON at all examined timepoints (5, 15, 30, and 60 min; *P* < 0.05; Figure 7). In contrast, the SBC5-3 group alone did not significantly alter the p-IκBα/IκBα ratio at these timepoints (*P* > 0.05). Co-treatment with SBC5-3 + TNF-α group exhibited a decrease in the p-IκBα/IκBα ratio compared to TNF-α alone at all-time points (5, 15, 30, and 60 min), with statistically significant suppression observed at 30 min and 60 min (*P* < 0.05). These data demonstrate that TNF-α induces both IκBα degradation and phosphorylation in HT-29 cells, while 16-h pre-incubation with *L. reuteri* SBC5-3 attenuates these effects by preserving IκBα expression and decreasing its phosphorylation level.

## 4 Discussion

The gastrointestinal tract serves as the principal immunological interface in vertebrates, critically regulating systemic immune homeostasis (Vancamelbeke and Vermeire, 2017). Modern livestock production systems, characterized by high stocking densities and frequent pathogen exposure, compromise intestinal immune competence, with consequent reductions in growth performance and economic returns from meat and egg production (Celi et al., 2017). The global restriction on AGPs has exacerbated this challenge, as existing nutritional strategies show insufficient capacity to alleviate diet-induced enteric inflammation and microbial dysbiosis (Shao et al., 2021; Wang et al., 2023). This immunological dysfunction triggers a pathogenic cascade marked by persistent mucosal inflammation, elevated pro-inflammatory cytokine expression, and progressive impairment of intestinal barrier function. These pathophysiological changes collectively undermine both production efficiency and animal welfare standards (Small et al., 2013; Teodorowicz et al., 2018). Current research reveals distinct strain-specific immunomodulatory properties of LAB, demonstrating functional divergence between pro-inflammatory and anti-inflammatory activities (Vincenzi et al., 2021). Certain strains enhance immune responses through cytokine induction, suggesting potential therapeutic applications for immunodeficiency disorders (Rocha-Ramírez et al., 2017). Conversely, other LAB strains predominantly mediate immunosuppressive effects through multiple pathways. Specifically, *L. plantarum* Lp01 exhibited bifunctional immunoregulation in *Salmonella*-infected murine models, concurrently inhibiting pro-inflammatory cytokines (including TNF-α, IL-6) while stimulating IL-10 production (Liu et al., 2023). Analogously, *L. rhamnosus* CY12 ameliorated intestinal inflammation through modulation of the TLR4-MyD88-NF-κB signaling pathway, leading to elevated IL-10 levels accompanied by marked decreases in IL-1β and TNF-α levels (Zheng et al., 2024). These findings highlight the importance of strain-level characterization of LAB–epithelial interactions, which proves essential for designing targeted microbiota therapies with defined immunoregulatory outcomes.

TNF-α, a pivotal pro-inflammatory cytokine, mediates both cell proliferation or induces apoptosis, playing crucial roles in inflammation and immunity (Zelová and Hošek, 2013). In HT-29 cells, TNF-α stimulation markedly elevates IL-8 production, a well-established biomarker for inflammatory model development (Sharma et al., 2007; Noh et al., 2013). Previous study (Bao et al., 2023) demonstrated that *L. reuteri* SBC5-3 co-culturing HT-29 cells substantially decreases TNF-α-induced *IL-8* gene expression, revealing its immunomodulatory capacity. To further elucidate the underlying mechanisms, RNA-seq was performed to analyze gene expression changes in HT-29 cells following TNF-α stimulation, both in the presence and absence of *L. reuteri* SBC5-3 co-culture, thereby elucidating how *L. reuteri* SBC5-3 modulates this inflammatory response.

In the absence of TNF-α stimulation, *L. reuteri* SBC5-3 fails to trigger pro-inflammatory gene expression in HT-29 cells. However, TNF-α treatment induces differential expression of 27 genes in HT-29 cells, including upregulation of 14 genes functionally linked to TNF and NF-κB signaling pathways. Following *L. reuteri* SBC5-3 intervention, nine of these upregulated genes (*BIRC3*, *PTGS2*, *CCL20*, *TNFAIP3*, *LTB*, *CXCL1*, *CXCL10*, *IL-8*, and *CSF1*) exhibited significant downregulation. This observation aligns with previous studies. For instance, a study (Lin et al., 2016) reported that treating HT-29 cells with *L. helveticus*-fermented soybean extract decreased the expression of genes including *CXCL1*, *CXCL2*, *CXCL3*, *LTB*, *IL-8*, *CSF1*, and *BIRC3*, which TNF-α induced. Consistent with these results, *L. fermentum* 664 was shown to downregulate expression of *TNF-*α, *CXCL6*, *CXCL1*β, and *PTGS2* in LPS-stimulated RAW264.7 macrophages (Hao et al., 2024). Chemokines including *CXCL1, CXCL10, CCL20*, and *IL-8* demonstrate characteristic upregulation during inflammation, facilitating the recruitment of neutrophils and T cells to the affected tissues (Laing and Secombes, 2004). CSF1 stimulates monocyte and macrophage proliferation and differentiation, thereby amplifying immune response (Sehgal et al., 2021). PTGS2 catalyzes prostaglandins synthesis, generating lipid mediators that contribute substantially to inflammatory processes (Ricciotti and FitzGerald, 2011). LTB functions as a potent inflammatory mediator through binding to the lymphotoxin-beta receptor, which triggers NF-κB signaling pathwayactivation and subsequent pro-inflammatory genes transcription (Dejardin et al., 2002; Li et al., 2020). The observed downregulation of these critical inflammatory markers indicates that *L. reuteri* SBC5-3 potentially attenuates TNF-α-induced cellular inflammation via three distinct mechanisms: modulating immune cell recruitment and activation, reducing prostaglandin synthesis, and suppressing NF-κB signaling pathways. Notably, TNFAIP3 (A20) and BIRC3, NF-κB-responsive genes encoding negative feedback regulators, exhibited downregulation following SBC5-3 treatment. Although this observation appears paradoxical given their anti-inflammatory functions, the reduced expression indicates diminished NF-κB activation, as sustained pathway activity is necessary to maintain their transcription levels (Momtazi et al., 2019; Frazzi, 2021). This finding correlates with the observation of suppressed IκBα phosphorylation, thereby confirming NF-κB signaling inhibition.

The NF-κB signaling pathway represents a pivotal regulator of inflammation responses due to its central role in controlling inflammation-related gene expression, rapid activation kinetics, extensive biological effects, and cross-talk with other signaling pathways (Liu et al., 2017). Treatment with *L. reuteri* SBC5-3 downregulated multiple established direct targets of the NF-κB, including *IL-8*, *CXCL10*, *BIRC3*, *TNFAIP3*, and *PTGS2*. Additional genes affected by this treatment may modulate NF-κB signaling through indirect mechanisms. KEGG enrichment analysis revealed significant enrichment of the NF-κB signaling pathway across all comparison groups, indicating that *L. reuteri* SBC5-3 may regulate the NF-κB pathway through unique mechanisms.

The canonical NF-κB pathway is primarily regulated through a core signaling axis comprising TAK1, IKKβ, and IκBα. Initial phosphorylation of TAK1 at Thr184/187 triggers a sequential activation cascade leading to IKKβ activation phosphorylated at Ser177/181 and eventual IκBα degradation (Karin, 1999). Within TNFR1 signaling, ligand binding induces the formation of a RIPK1-TRAF2/5 complex, which mediates K63-linked polyubiquitination of RIPK1. This ubiquitin scaffold recruits the TAK1-TAB2/3 complex, enabling TAK1 autophosphorylation and kinase activation (Hacker and Karin, 2006). The activated TAK1 directly phosphorylates IKKβ, which mediates IκBα phosphorylation at Ser32/36. This phosphorylation event targets IκBα for K48-linked ubiquitination and proteasomal degradation, ultimately liberating the p50/p65 heterodimer for nuclear translocation and transcriptional activation of pro-inflammatory mediators (Hayden and Ghosh, 2004).

The current study revealed that *L. reuteri* SBC5-3 treatment markedly reduced expression levels of RIPK1, TRAF5, TAK1, IKKα, and NFKB1 in TNF-α-stimulated HT-29 cells. These observations indicate potential interference with TRAF2/5-RIPK1 signaling complex formation at the upstream region of the NF-κB pathway, resulting in inhibition of K63-linked polyubiquitination, an essential step for TAK1 phosphorylation and activation. Such disruption led to subsequent suppression of IKKβ phosphorylation and IκBα degradation, thereby enabling the blockade of nuclear translocation of the p50/p65 heterodimer. Furthermore, SBC5-3-mediated transcriptional downregulation of *TAK1*, *IKK*α, and *NFKB1* may promote prolonged pathway suppression through depletion of these critical signaling components.

Western blot analysis revealed that *L. reuteri* SBC5-3 significantly suppressed phosphorylation of TAK1, IKKα/β, and IκBα in TNF-α-induced HT-29 cells, consequently mitigating the inflammatory response induced by TNF-α. The current observations align with previously reported findings. Specifically, Li et al. (2022) documented that *L. plantarum* 17-5 mitigated the inflammation induced by *Escherichia coli* in mammary epithelial cells by inhibiting IκBα phosphorylation. Similarly, Lee et al. (2015) observed that *L. sakei* OK67 alleviated collagen-induced arthritis in murine models via suppression of both TAK1 phosphorylation and NF-κB activation. Additionally, Chen et al. (2021) establishedthat *L. plantarum* KLDS 1.0344 diminished inflammatory cytokine expression in LPS-challenged mouse mammary epithelial cells by blocking p65 and IκBα phosphorylation. Our research demonstrates that immune regulation mediated by *L. reuteri* SBC5-3 involves the inhibition of IκBα degradation, a process essential for the nuclear translocation of p50/p65 and subsequent DNA binding (Ferreiro and Komives, 2010). This finding suggests that SBC5-3 may inhibit the nuclear translocation of the p50/p65 dimer, thereby modulating the inflammatory response. However, this hypothesis lacks direct experimental support. Future studies will employ direct methods, such as p65 immunofluorescence, to verify this mechanism.

Recent studies have documented that LAB can modulate host immune response through multiple mechanisms (Vincenzi et al., 2021). In the present investigation, *L. reuteri* SBC5-3 treatment markedly reduced *JNK* and *ERK* genes expression in TNF-α-induced inflammation model using HT-29 cells. As critical kinases within the MAPK pathway, JNK and ERK serve crucial functions in transducing signals that lead to the activation and nuclear translocation of transcription factors responsible for pro-inflammatory cytokine gene expression (Papa et al., 2019). These findings suggest that *L. reuteri* SBC5-3 potentially modulates immune response in HT-29 cells through suppression of these kinase genes.

As demonstrated in this study, *L. reuteri* SBC5-3 effectively blocked TNF-α-induced TAK1 phosphorylation. TAK1 functions as an upstream kinase that activates both NF-κB and MAPK signaling pathways upon phosphorylation. The observed inhibition of TAK1 phosphorylation may interfere with the upstream activation of the MAPK pathway, thereby suppressing the downstream phosphorylation of MAPK family members, including ERK, JNK, and p38 (Mihaly et al., 2014). Such inhibition may ultimately decrease pro-inflammatory cytokines transcription, alleviating inflammation and cellular damage triggered by external stimuli. Additionally, analysis revealed significant downregulation of *TLR4* gene expression in both SBC5-3 + TNF-α versus TNF-α and SBC5-3 versus CON comparison groups. Similar effects were reported for *Bifidobacterium infantis* ATCC 15697 (Meng et al., 2016) and *L. rhamnosus* CCFM1120 (Niu et al., 2024). TLR4 serves as a core innate immune system receptor primarily responsible for recognizing LPS from Gram-negative bacteria. Upon activation, this receptor initiates downstream NF-κB and MAPK signaling cascades (Zamyatina and Heine, 2020). The observed reduction in *TLR4* expression suggests potential therapeutic benefits for modulating Gram-negative bacteria-induced inflammatory responses.

The immunomodulatory effects of *L. reuteri* SBC5-3 may be associated with metabolites such as lipoteichoic acid, acetate, and histamine. Current studies indicate that lipoteichoic acid from *L. paracasei* 6-1 suppresses LPS-induced inflammatory responses in RAW264.7 cells by inhibiting the TLR4-MyD88-MAPK and NF-κB signaling pathways (Zhang et al., 2023). Acetate can inhibit LPS-induced NF-κB activation in RAW264.7 cells and reduce the production of pro-inflammatory cytokines such as TNF-α, IL-1β, and IL-6 (Liu et al., 2012). Histamine produced by *L. reuteri* ATCC PTA 6475 can suppress TNF production in a Pam3Cys-SKKKK-induced THP-1 cell inflammation model by modulating PKA and ERK signaling (Thomas et al., 2012). Genomic analysis of *L. reuteri* SBC5-3 reveals the absence of critical histamine biosynthesis genes, specifically the hdc gene cluster (*hdcA*, *hdcB*, and *hdcP*) (unpublished data), suggesting that its anti-inflammatory properties may operate through histamine-independent mechanisms. These findings highlight the need for subsequent investigations to evaluate the immunomodulatory potential of *L. reuteri* SBC5-3-derived lipoteichoic acid and metabolic byproducts, which could elucidate the strain-specific anti-inflammatory pathways.

## 5 Conclusion

The anti-inflammatory mechanism of *L. reuteri* SBC5-3 primarily involves transcriptional suppression of key regulatory genes in the NF-κB signaling pathway, including *TAK1*, *IKK*α, and *NFKB1.* This regulatory effect extends to phosphorylation inhibition of TAK1, IKKα/β, and IκBα, consequently disrupting the NF-κB signaling cascade by preventing both IκBα degradation. Furthermore, *L. reuteri* SBC5-3 appears to suppress ERK and JNK transcription in the MAPK signaling pathway while reducing TAK1 phosphorylation, thereby potentially interfering with the p38/ERK/JNK-mediated MAPK cascade. These findings provide a theoretical foundation for developing novel microbiome-based therapeutics utilizing *L. reuteri* SBC5-3.

## Data Availability

The raw data associated with this study are accessible through the NCBI Sequence Read Archive (SRA) database under BioProject ID PRJNA1221070. The whole-genome sequence data of *Lactobacillus reuteri* SBC5-3 are also accessible through the NCBI database under BioProject ID PRJNA1233652.
