# Supplementary figures and images for: Lactobacillus reuteri SBC5-3 suppresses TNF-α-induced inflammatory responses via NF-κB pathway inhibition in intestinal epithelial cells
Source: Front Microbiol. 2025 Jul 8;16:1573479. doi: 10.3389/fmicb.2025.1573479 (PMC12279514; doi:10.3389/fmicb.2025.1573479)

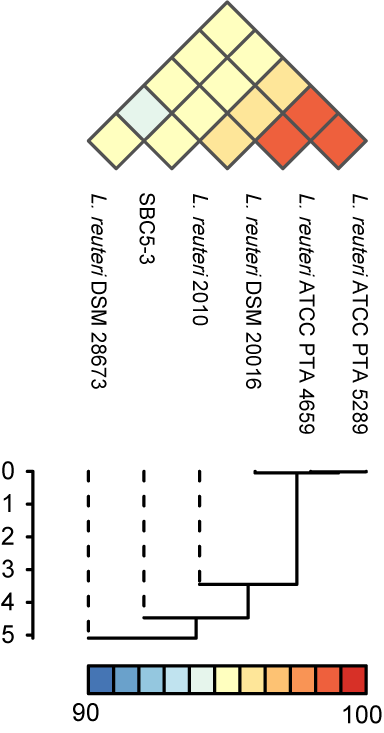


**Figure S1. ANI Analysis of Strain SBC5-3 with Five Representative** *Lactobacillus reuteri* **Strains**

Supplement: Supplementary file 1 [file Data_Sheet_1.docx]
