# Supplementary material for: Lactobacillus reuteri SBC5-3 suppresses TNF-α-induced inflammatory responses via NF-κB pathway inhibition in intestinal epithelial cells
Source: Front Microbiol. 2025 Jul 8;16:1573479. doi: 10.3389/fmicb.2025.1573479 (PMC12279514; doi:10.3389/fmicb.2025.1573479)
Supplement: Supplementary file 2 [file Table_1.docx]

Table S1. Primers for Quantitative Real-time PCR.

| Gene name | Primer sequences (5'-3') |
| --- | --- |
| *ACTB* | F: TTGTTACAGGAAGTCCCTTGCC  R: ATGCTATCACCTCCCCTGTGTG |
| *IL-8* | F: GAATGGGTTTGCTAGAATGTGATA  R: CAGACTAGGGTTGCCAGATTTAAC |
| *IL-1β* | F: GTGGCAATGAGGATGACTTGTTC  R: TTGCTGTAGTGGTCGGAG |
| *CCL20* | F: CTGCTTTGATGTCAGTGCTGCTAC  R: CTGCCGTGTGAAGCCCACAATAAA |
| *CXCL10* | F: CCAGAATCGAAGGCCATCAA  R: CATTTCCTTGCTAACTGCTTTCAG |
| *NFKB1* | F: GCAGCACTACTTCTTGACCACC  R: TCTGCTCCTGAGCATTGACGTC |
| *NFKBIA* | F: CTCCGAGACTTTCGAGGAAATAC  R: GCCATTGAAGTTGGTAGCCTTCA |
| *PTGS2* | F: CTGGCGCTCAGCCATACAG  R: CGCACTTATACTGGTCAAATCCC |
| *TNFAIP3* | F: CTTGTGGCGCTGAAAACGAA  R: CCACTGTCCTTCAGGGTCAC |
