# Supplementary material for: Lactobacillus reuteri SBC5-3 suppresses TNF-α-induced inflammatory responses via NF-κB pathway inhibition in intestinal epithelial cells
Source: Front Microbiol. 2025 Jul 8;16:1573479. doi: 10.3389/fmicb.2025.1573479 (PMC12279514; doi:10.3389/fmicb.2025.1573479)
Supplement: Supplementary file 3 [file Table_2.docx]

Table S2. Effect of SBC5-3 on MAPK Signaling Pathway Gene Expression in TNF-α-Induced HT-29 Cells

| Gene name | Gene description | Log2 (Fold change) |
| --- | --- | --- |
| *HSPA6* | heat shock protein family A (Hsp70) member 6 | 9.91 |
| *DDIT3* | DNA damage-inducible transcript 3 | 4.11 |
| *DUSP1* | dual specificity phosphatase 1 | 3.99 |
| *GADD45B* | Growth arrest and DNA damage-inducible protein GADD45 beta | 3.40 |
| *FOS* | proto-oncogene protein c-fos | 2.87 |
| *MKNK2* | MAPK interacting serine/threonine kinase 2 | 2.42 |
| *JUND* | transcription factor jun-D | 2.36 |
| *NR4A1* | nuclear receptor subfamily 4 group A member 1 | 2.31 |
| *HSPB1* | heat shock protein beta-1 | 2.07 |
| *ECSIT* | Evolutionarily conserved signaling intermediate in Toll pathway | 1.98 |
| *JUN* | transcription factor AP-1 | 1.88 |
| *MAP2K2* | mitogen-activated protein kinase kinase 1 | 1.83 |
| *RAC3* | ras-related C3 botulinum toxin substrate 3 | 1.66 |
| *MAPK11* | mitogen-activated protein kinase 11 | 1.55 |
| *MAP3K11* | mitogen-activated protein kinase kinase kinase 11 variant | 1.50 |
| *CDC25B* | M-phase inducer phosphatase 2 | 1.48 |
| *MAPKAPK3* | mitogen-activated protein kinase-activated protein kinase 3 | 1.44 |
| *ELK1* | ETS domain-containing protein Elk-1 | 1.37 |
| *RELA* | transcription factor p65 | 1.27 |
| *MAPK7* | Mitogen activated protein kinase 7 transcript variant 5 variant | 1.23 |
| *RPS6KA4* | ribosomal protein S6 kinase alpha-4 | 1.18 |
| *ARAF* | serine/threonine-protein kinase A-Raf isoform 2 | 1.17 |
| *MAP3K14* | mitogen-activated protein kinase kinase kinase 14 | 1.16 |
| *IRAK1* | Interleukin-1 receptor-associated kinase 1 | 1.14 |
| *TAB1* | TGF-beta-activated kinase 1 and MAP3K7-binding protein 1 | 1.07 |
| *SRF* | serum response factor | 1.04 |
| *ATF4* | activating transcription factor 4 | 1.04 |
| *TRAF2* | TNF receptor-associated factor 2 | 1.01 |
| *CDC42* | cell division control protein 42 | -1.06 |
| *JNK* | mitogen-activated protein kinase 8 | -1.44 |
| *MAP2K4* | mitogen-activated protein kinase kinase 4 | -1.13 |
| *TAOK3* | serine/threonine-protein kinase TAO3 | -1.15 |
| *ERK* | mitogen-activated protein kinase 1 | -1.23 |
| *NLK* | nemo like kinase | -1.25 |
| *ATF2* | cyclic AMP-dependent transcription factor ATF-2 | -1.27 |
| *IRAK4* | Interleukin-1 receptor-associated kinase 4 | -1.27 |
| *MAP3K2* | mitogen-activated protein kinase kinase kinase 2 | -1.34 |
| *PLA2G4A* | phospholipase A2 group IVA | -1.36 |
| *MAP3K4* | Mitogen-activated protein kinase kinase kinase 4 | -1.36 |
| *MAP3K1* | mitogen-activated protein kinase kinase kinase 1 | -1.44 |
| *CASP3* | caspase-3 | -1.51 |
| *RASGRF2* | ras-specific guanine nucleotide-releasing factor 2 | -1.52 |
| *MAP3K7* | mitogen-activated protein kinase kinase kinase 7 isoform B | -1.53 |
| *MYC* | Myc proto-oncogene protein | -1.53 |
| *RPS6KA3* | ribosomal protein S6 kinase A3 | -1.54 |
| *NF1* | neurofibromin 1 | -1.58 |
| *IKKA* | inhibitor of nuclear factor kappa-B kinase subunit alpha | -1.65 |
| *ELK4* | ETS domain-containing protein Elk-4 | -1.77 |
| *NFATC3* | nuclear factor of activated T cells 3 | -1.79 |
| *RASA1* | Ras GTPase-activating protein 1 | -1.94 |
| *RASA2* | Ras GTPase-activating protein 2 | -2.24 |
| *MAP3K5* | mitogen-activated protein kinase kinase kinase 5 variant | -2.44 |
| *PRKACB* | cAMP-dependent protein kinase catalytic subunit beta isoform 2 | -2.70 |
