# Supplementary material for: Lactobacillus reuteri SBC5-3 suppresses TNF-α-induced inflammatory responses via NF-κB pathway inhibition in intestinal epithelial cells
Source: Front Microbiol. 2025 Jul 8;16:1573479. doi: 10.3389/fmicb.2025.1573479 (PMC12279514; doi:10.3389/fmicb.2025.1573479)
Supplement: Supplementary file 4 [file Table_3.docx]

Table S3. Effect of SBC5-3 on MAPK Signaling Pathway Gene Expression in HT-29 Cells

| Gene name | Gene description | Log2 (Fold change) |
| --- | --- | --- |
| *HSPA6* | Heat shock 70 kDa protein 6 | 9.52 |
| *DDIT3* | DNA damage-inducible transcript 3 protein | 4.34 |
| *DUSP1* | Dual specificity protein phosphatase 1 | 4.07 |
| *GADD45B* | Growth arrest and DNA damage-inducible protein GADD45 beta | 3.01 |
| *FOS* | Proto-oncogene c-Fos | 2.74 |
| *JUND* | Transcription factor jun-D | 2.47 |
| *MKNK2* | MAP kinase-interacting serine/threonine-protein kinase 2 | 2.47 |
| *JUN* | Transcription factor AP-1 | 2.20 |
| *NR4A1* | Nuclear receptor subfamily 4 group A member 1 | 2.12 |
| *MAPK3* | Mitogen-activated protein kinase 3 | 1.92 |
| *HSPB1* | Heat shock protein beta-1 | 1.83 |
| *EREG* | Proepiregulin | 1.82 |
| *DUSP4* | Dual specificity protein phosphatase 4 | 1.74 |
| *NFKB2* | Nuclear factor NF-kappa-B p100 subunit | 1.60 |
| *MAP2K2* | Dual specificity mitogen-activated protein kinase kinase 2 | 1.60 |
| *ECSIT* | Evolutionarily conserved signaling intermediate in Toll pathway, mitochondrial | 1.60 |
| *RAC3* | Ras-related C3 botulinum toxin substrate 3 | 1.55 |
| *TNFRSF1A* | Tumor necrosis factor receptor superfamily member 1A | 1.53 |
| *MAP4K2* | Mitogen-activated protein kinase kinase kinase kinase 2 | 1.42 |
| *MAP3K11* | Mitogen-activated protein kinase kinase kinase 11 | 1.36 |
| *CDC25B* | M-phase inducer phosphatase 2 | 1.35 |
| *SRF* | Serum response factor | 1.33 |
| *HRAS* | GTPase HRas | 1.28 |
| *ELK1* | ETS domain-containing protein Elk-1 | 1.26 |
| *ATF4* | Cyclic AMP-dependent transcription factor ATF-4 | 1.23 |
| *TRADD* | Tumor necrosis factor receptor type 1-associated DEATH domain protein | 1.23 |
| *ARRB2* | Beta-arrestin-2 | 1.18 |
| *MAPKAPK3* | MAP kinase-activated protein kinase 3 | 1.15 |
| *MAPK7* | Mitogen-activated protein kinase 7 | 1.11 |
| *CASP3* | Caspase-3 | -1.01 |
| *MAP4K3* | Mitogen-activated protein kinase kinase kinase kinase 3 | -1.02 |
| *RAPGEF2* | Rap guanine nucleotide exchange factor 2 | -1.03 |
| *IRAK4* | Interleukin-1 receptor-associated kinase 4 | -1.03 |
| *NF1* | Neurofibromin | -1.08 |
| *MET* | Hepatocyte growth factor receptor | -1.09 |
| *GNG12* | Guanine nucleotide-binding protein G(I)/G(S)/G(O) subunit gamma-12 | -1.11 |
| *SOS1* | Son of sevenless homolog 1 | -1.14 |
| *NFKB1* | Nuclear factor NF-kappa-B p105 subunit | -1.14 |
| *CRK* | Adapter molecule crk | -1.15 |
| *MAP3K4* | Mitogen-activated protein kinase kinase kinase 4 | -1.18 |
| *MAP3K1* | Mitogen-activated protein kinase kinase kinase 1 | -1.27 |
| *RPS6KA3* | Ribosomal protein S6 kinase alpha-3 | -1.33 |
| *JNK* | Mitogen-activated protein kinase 8 | -1.36 |
| *TAOK1* | Serine/threonine-protein kinase TAO1 | -1.42 |
| *STK4* | Serine/threonine-protein kinase 4 | -1.44 |
| *MYC* | Myc proto-oncogene protein | -1.49 |
| *CHUK* | Inhibitor of nuclear factor kappa-B kinase subunit alpha | -1.53 |
| *MECOM* | MDS1 and EVI1 complex locus protein | -1.53 |
| *ELK4* | ETS domain-containing protein Elk-4 | -1.55 |
| *MAP3K7* | Mitogen-activated protein kinase kinase kinase 7 | -1.57 |
| *PLA2G4A* | Cytosolic phospholipase A2 | -1.57 |
| *PPP3CA* | Serine/threonine-protein phosphatase 2B catalytic subunit alpha isoform | -1.59 |
| *RASGRF2* | Ras-specific guanine nucleotide-releasing factor 2 | -1.61 |
| *TGFBR1* | TGF-beta receptor type-1 | -1.70 |
| *HSPA2* | Heat shock-related 70 kDa protein 2 | -1.77 |
| *RASA1* | Ras GTPase-activating protein 1 | -1.80 |
| *MAP3K5* | Mitogen-activated protein kinase kinase kinase 5 | -1.88 |
| *TGFB2* | Transforming growth factor beta-2 | -2.04 |
| *PRKACB* | cAMP-dependent protein kinase catalytic subunit beta | -2.05 |
| *NFATC3* | Nuclear factor of activated T-cells, cytoplasmic 3 | -2.23 |
| *IL1R1* | Interleukin-1 receptor type 1 | -2.38 |
